# Genomic and Chemical Diversity of Commercially Available High-CBD Industrial Hemp Accessions

**DOI:** 10.3389/fgene.2021.682475

**Published:** 2021-07-07

**Authors:** Matthew S. Johnson, Jason G. Wallace

**Affiliations:** ^1^Institute of Plant Breeding, Genetics, and Genomics, University of Georgia, Athens, GA, United States; ^2^Department of Crop and Soil Sciences, University of Georgia, Athens, GA, United States

**Keywords:** hemp (*Cannabis sativa* L), cannabidiol (CBD), tetrahydrocannabinol (THC), cannabinoid, genetic diversity

## Abstract

High consumer demand for cannabidiol (CBD) has made high-CBD hemp (*Cannabis sativa*) an extremely high-value crop. However, high demand has resulted in the industry developing faster than the research, resulting in the sale of many hemp accessions with inconsistent performance and chemical profiles. These inconsistencies cause significant economic and legal problems for growers interested in producing high-CBD hemp. To determine the genetic and phenotypic consistency in available high-CBD hemp varieties, we obtained seed or clones from 22 different named accessions meant for commercial production. Genotypes (∼48,000 SNPs) and chemical profiles (% CBD and THC by dry weight) were determined for up to 8 plants per accession. Many accessions–including several with the same name–showed little consistency either genetically or chemically. Most seed-grown accessions also deviated significantly from their purported levels of CBD and THC based on the supplied certificates of analysis. Several also showed evidence of an active tetrahydrocannabinolic acid (THCa) synthase gene, leading to unacceptably high levels of THC in female flowers. We conclude that the current market for high-CBD hemp varieties is highly unreliable, making many purchases risky for growers. We suggest options for addressing these issues, such using unique names and developing seed and plant certification programs to ensure the availability of high-quality, verified planting materials.

## Introduction

Hemp (*Cannabis sativa)* is a dioecious annual plant that is thought to have been domesticated around 6,000 years ago in China, with some evidence of use as far back as 12,000 years ago (Li, 1974; Fleming and Clarke, 1998; Merlin, 2003). Different hemp varieties have been used for fiber, seeds, medicine, and recreation for thousands of years (Russo, 2007). Hemp has also recently been used to produce biofuels (Li et al., 2010), plastics (Wretfors et al., 2009; Khattab and Dahman, 2019), and building composites (Sassoni et al., 2014; Hussain et al., 2019).

Similar to other crops, different hemp varieties serve specific uses. However, until recently the United States had banned all *C. sativa* varieties from commercial production due some of them being used as recreational drugs (marijuana) (Alliance, 2014). The psychoactive properties of marijuana are due to high amounts of a specific secondary metabolite, tetrahydrocannabinol (THC). Recognizing that not all hemp is created equal, the 2018 United States Farm Bill allowed growers to cultivate “industrial” hemp (defined as varieties with < 0.3% THC by dry weight) throughout the United States (S. 2667 (115th): Hemp farming act of 2018, 2018). This has led to a surge of interest in hemp production, especially for varieties with high production of other, non-psychoactive metabolites (cannabinoids). Currently, the largest interest is in varieties bred to produce cannabidiol (CBD), a non-psychoactive cannabinoid used as a medicine and health food supplement. Both CBD and THC derive from the same precursor, cannabigerolic acid (CBGa) (Sirikantaramas et al., 2004; Taura et al., 2007). They are most concentrated in the trichomes of female flowers, as are the over 100 other known cannabinoids present at much lower concentrations (Turner et al., 1981; Elsohly and Slade, 2005).

The most well-studied application of CBD is to control seizures, which is the basis of the FDA-approved drug epidiolex. CBD is also marketed as a nutritional supplement to help with anxiety, pain, depression, and sleep; most of these claims are anecdotal, although there are some studies supporting them (Perucca, 2017; Glass and Gilleece, 2019; Hurd et al., 2019). Regardless of efficacy, the market value of CBD products is currently estimated at over $4.7 billion dollars per year (2020 US CBD market forecast announcement, 2020).

### Hemp Genetics

*Cannabis sativa* has a diploid genome (2n = 20) with an estimated size of 818 Mb for female plants and 843 Mb for males (Sakamoto et al., 1998). Since pollination lowers cannabinoid yield by ∼50% (Meier and Mediavilla, 1998), growers interested in these compounds often use “feminized” seed or clones of female plants. [Feminized seed is produced from two genetically female plants, one of which has been chemically treated to produce male flowers (Ram et al., 1972; Lubell and Brand, 2018).]

Until recently, hemp’s legal status prevented most research on it. Lack of research made it essentially an orphan crop, with few genomic resources and almost no public germplasm collections. Despite these restrictions, there have been significant advances in understanding hemp genetics for several key traits, including cannabinoid production (Taura et al., 1995; de Meijer et al., 2003, 2009; Weiblen et al., 2015), sex expression (Faux et al., 2016), fiber quality (van den Broeck et al., 2008), and population structure and diversity (Sawler et al., 2015; Lynch et al., 2016; Dufresnes et al., 2017). Many more genomics resources have become available for hemp over the past decade, including multiple genome sequences (van Bakel et al., 2011; Laverty et al., 2019), transcriptomes analyses (Liu et al., 2016; Braich et al., 2019; Huang et al., 2019; McGarvey et al., 2020), and proteome analyses (Jenkins and Orsburn, 2020; Conneely et al., 2021). These resources are rapidly bringing hemp into the genomics era and ending its status as an orphan crop.

Due to a long history of breeding for different purposes, drug-type *C. sativa* plants form genetically distinct clusters from fiber and grain types (van Bakel et al., 2011; Sawler et al., 2015; Lynch et al., 2016; Vergara et al., 2021). Prior studies usually focused on marijuana, but sometimes included high CBD/low THC varieties as well. Perhaps not surprisingly, high-CBD varieties are closely related to marijuana varieties (Grassa et al., 2021) since both types have been bred for production of specific secondary metabolites (cannabinoids and terpenoids, etc.).

### Issues With Commercial Hemp Cultivation

Although interest in commercial hemp cultivation has exploded since the 2018 Farm Bill, many issues of naming and quality control plague the field. Because all varieties of hemp were outlawed for several decades, breeding and naming of varieties has been largely clandestine and *ad hoc*, with names frequently recycled to reflect the most successful or desirable cultivars. Thus there is no guarantee that the variety “Cherry Wine” received from one supplier is the same as–or even related to–a variety of the same name from a different supplier. For example, Both Lynch et al. (2016) and Schwabe et al. (2019) found that the traditional classifications of “indica” and “sativa” for drug-type *Cannabis* did not reflect their genetic relationships, and that high-CBD/low THC varieties generally cluster separately from drug-type marijuana plants. The high-CBD reference genome, CBDRx, is an exception, sharing 89% of its genome with marijuana varieties (Grassa et al., 2021). Marijuana-type *Cannabis* is also known to have significant naming inconsistencies, where multiple plants with the same name are actually genetically distinct (Sawler et al., 2015; Schwabe and McGlaughlin, 2019). This inconsistency includes not just background genetics but also how many copies of the cannabinoid biosynthesis genes are present (Vergara et al., 2019), arguably the most important trait for these varieties.

In addition to names, standards for CBD and THC production are lacking. This is particularly important because any plants with >0.3% THC are classified as marijuana and must be destroyed, causing significant loss of revenue. To increase grower confidence, some companies provide certificates of authenticity (COAs) that attest to how much of each compound a variety will produce.

Although *Cannabis* genetics has been developing quickly, the major focus is usually on marijuana-type *C. sativa*, with CBD-type hemp often much less represented (e.g., Lynch et al., 2016; Vergara et al., 2021). To date, there have been no studies that focus on the consistency of high-CBD hemp from the point of view of a commercial grower. We aimed to fill this gap by specifically investigating if the naming issues found in drug-type *C. sativa* also occurred in CBD-type varieties available for large-scale commercial production. To this end, we developed genetic and chemical diversity data sets on twenty-two commercially available hemp accessions. We identified both the genetic relationships among the accessions and the genetic consistency within each accession. We also tested the production of total CBD and THC for each line and compared these to industry and legal standards and the provided Certificates of Authenticity. These comparisons are not meant to evaluate specific sources or accessions *per se*, but rather to demonstrate the overall state of the market and give an idea of how reliable (or not) it is for interested growers.

## Materials and Methods

### Plant Material

Twenty-two commercial hemp accessions were purchased or donated from various sources (Table 1). Since accessions frequently pass among groups, it is impossible to say if these companies are the original sources, or if they have done any selections to differentiate them from the original source. This collection focused on cannabidiol (CBD) production, but accessions for fiber and seed were also included. Twenty of the accessions were distributed as seeds (some feminized, others not) and two of them were clonally propagated. For our experiments, all clonal plants were propagated from a single original plant to ensure that each replicate was an exact genetic copy.

**TABLE 1:** Accessions used in this study.

**Source**	**Accession**	**Use**
Blank land botanicals (https://blacklandsbotanicals.org/)	AbacusxBB#1	CBD
	Berry Blossom	CBD
	C4	CBD
	C4xBB#1	CBD
	Cherry (original)	CBD
	Cherry Wine	CBD
	Otto II	CBD
	Wife	CBD
Bulk hemp warehouse (https://www.bulkhempwarehouse.com/)	BHWH_Chinese_Fiber_Hemp	Fiber
	BHWH_For_Fiber	Fiber
	GF_BHWH	Grain
Colorado CBD (https://www.coloradocbdseed.com/)	Abacus_Early_Bird	CBD
	Abacus_Early_Bird_2.0	CBD
Cross creek hemp (https://crosscreekhemp.com/)	BaoxSP_07	CBD
	LifterSP_01	CBD
Earth matters hemp (https://hawaiicannabis.org/earth-matters-farm/)	Ka’uXX	Fiber/CBD Cross
	Ka’uXXX	Fiber/CBD Cross
GaXtracts (https://www.gaxtracts.com/)	Baox	CBD
	Chardonnay	CBD
	Cherry/Wu	CBD
	Oregon Melon	CBD
	Otto	CBD

Seeds were soaked in water for 12 h to initiate germination. Due to low germination rates, 15 seeds were planted per half-gallon pot and thinned to only 1 plant per pot after 2 weeks. Clones were made by cutting a seven-inch section of stem from the mother plant, trimming off all leaves and growing points except the topmost one, and dipping in cloneX (Growth Technology) rooting solutions before planting into half-gallon pots. All plants were grown in a commercial potting medium (Sun Gro Metro Mix 830). Plants were fertilized twice a week using a diluted 20-20-20 fertilizer (1000 ppms) and a diluted micronutrient mixture (Jackpot Micronutrient Mixture; 500 ppm). To maintain the plants in a controlled vegetative state, growth conditions were kept under an 18-h light/6-h dark cycle. All plants were grown in greenhouses at the University of Georgia (Athens, GA, United States).

### Genomic Data

Ten leaf punches were taken from each plant and sent to LGC Genomics for DNA extraction and genotyping-by-sequencing (GBS) (Elshire et al., 2011) with restriction enzyme *Msl*I. GBS was chosen over shotgun sequencing due to the ability to get greater depth at sites, allowing us to accurately call heterozygous alleles. Paired-end 150 bp reads were generated using Illumina NextSeq V500/550. Libraries were demultiplexed using the Illumina bcl2fastq software (version 2.17.14). SNPs were aligned to the CBDRx reference genome [NCBI GCF_900626175.1; (Grassa et al., 2021)] with BWA mem (Li, 2013) and SNPs called with BCFtools (Li, 2011) requiring a minimum base quality of 20 and only outputting SNPs (not indels). All bioinformatic scripts (including exact parameters used) are available at https://github.com/wallacelab/paper-johnson-hemp-gs, and adaptor- and restriction-fragment-verified sequencing data is available at NCBI under Bioproject PRJNA707556.

Raw SNPs were then filtered in a series of steps. Misalignments and low-coverage sites were filtered out by removing all sites with an average genotyping depth of <15 reads per individual, and paralogs were removed by filtering out sites with >125 reads per individual. These cutoffs were based on initial data exploration that showed average genotyping depth to be ∼100 reads per individual (Supplementary Figure 1). We then removed sites present in < 80% of samples, with minor allele frequencies <2.5% (since most of these are sequencing errors) and with >10% heterozygosity (since these are often paralogs being misaligned to the same location). This resulted in 48,029 SNPs in the final dataset. Cladograms were generated using the neighbor-joining method in TASSEL v5.2.40 (Bradbury et al., 2007). For the neighbor-net analysis, TASSEL was also used to generate a genetic distance matrix, which was then processed with RSplitsTree (Bickel and Zakharko, 2016) and SpitsTree4 (Huson and Bryant, 2006).

To compare against prior work, public whole-genome sequencing data from 55 *C. sativa* varieties (Lynch et al., 2016) was downloaded from NCBI and SNPs called using the same pipeline as above, except that no depth filters were applied due to the much lower sequencing depth of this data (∼5×). We merged the two datasets by keeping only the sites present in both datasets after quality filtering, resulting in 8867 SNPs. Neighbor-net network analysis was performed as above.

### Cannabinoid Analysis

Fifty-two days after sowing, eight replicates of each accession were placed into a flower room with a 12-h light/12-h dark cycle to initiate flowering. Plants were laid out in a randomized complete block design. Any plants that showed male flowers were removed from the room to eliminate pollination. (All such plants were from non-feminized seed lots; Supplementary Table 1.) The remaining female plants were kept in the flowering room for 12 weeks, at which point the panicles in the top six inches of each plant were harvested, trimmed of excess leaf material, placed in a paper bag, and dried at 35°C for 2 weeks.

Cannabinoids levels were assayed with a Shimadzu LC2030C high-performance liquid chromatography (HPLC) machine according to the manufacturer’s recommended protocol. In brief, 200 mg of dried flower material was weighed out for each sample and the exact weight recorded. Samples were placed in 20 ml of methanol and agitated for 3 min to extract the cannabinoids. 1 ml of the supernatant was passed through a 0.22 um filter, and 50 μl of filtrate was diluted into 950 μl of methanol, resulting in a 400× dilution of the original samples. HPLC was carried out using a NexLeaf CBX column (2.7 μm, 4.6 × 150 mm; part number: 220-91525-70), NexLeaf CBX guard column (part number: 220-91525-72), eleven-cannabinoid standard mix (part number: 220-91239-21), and high sensitivity method solvents A (0.085% phosphoric acid in water) and B (0.085% phosphoric acid in acetonitrile) (part number: 220-91394-81). The flow rate was 1.5 ml/min with a gradient starting from 30% solvent/70% solvent B and ramping to 5% solvent A/95% solvent B over 8 min. Injection volume was 5 μL, and a guard column temperature of 35°C was maintained by an internal oven. Standard curves were generated for each target cannabinoid with minimum correlation coefficients (*R*^2^) of 0.999 over the six concentration levels (0.5, 1, 5, 10, 50, and 100 ppm). The original sample weights were used to determine the precise cannabinoid concentration in the original sample.

Genetic and chemical variation within accessions was compared by calculating the average genetic distance within each accession [calculated in TASSEL v5.2.58 (Bradbury et al., 2007)] and comparing it to the variance of measured CBD or THC for plants of that accession, using either raw or log-transformed data.

All figures were made in R using packages argparse v2.0.1 (Davis, 2019), ggplot2 v3.3.0 (Wickham, 2016), ggpubr v0.4.0 (Kassambara, 2020), ggtree v3.2 (Yu, 2020), gridExtra v2.3 (Auguie, 2017), RSplitsTree (Bickel and Zakharko, 2016), tidyverse (Wickham et al., 2019), treeio v1.14.4 (Wang et al., 2020), and vegan v2.5-7 (Oksanen et al., 2020).

## Results

We planted 8 replicates of the 22 accessions, resulting in 176 total pots. Of these, 3 had no seeds germinate despite having 15 seeds originally planted, and 26 developed male flowers and so were removed from the experiment. The males were from 11 different accessions, none of which claimed to be feminized seed; Supplementary Table 1).

**FIGURE 1:**
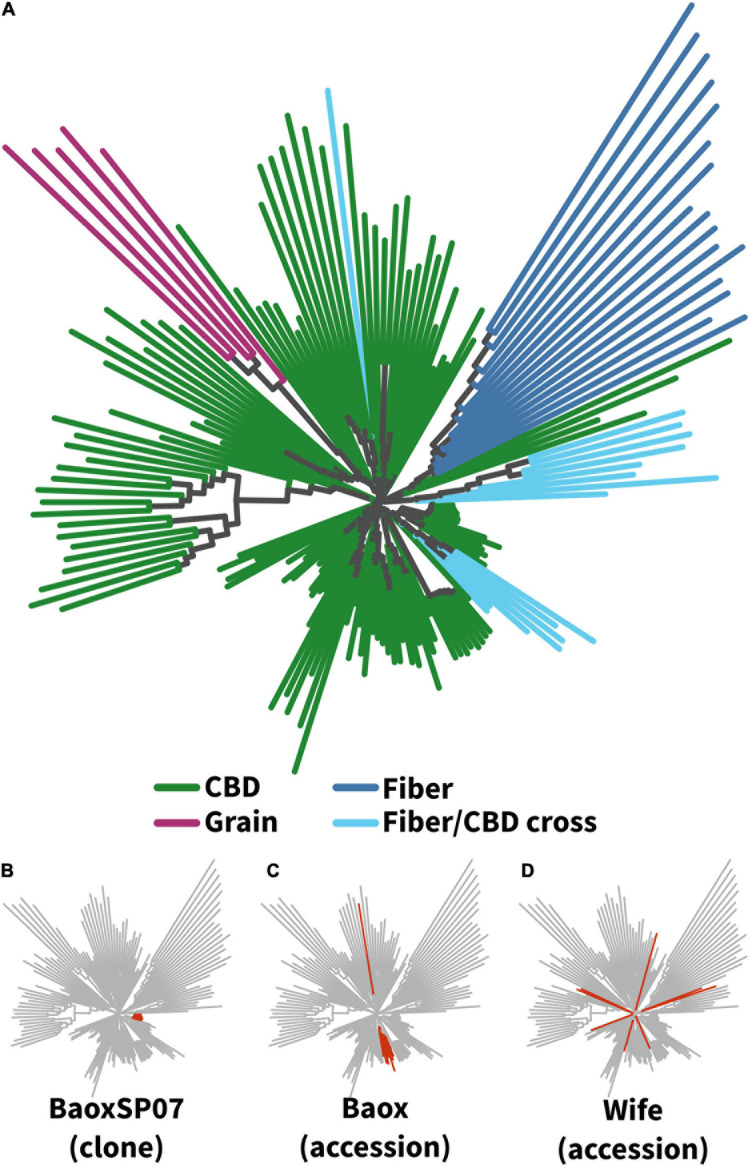
Relationships among accessions based on a neighbor-joining tree of SNP data. **(A)** All plants (173 plants from 22 accessions). Branch tips are colored by use type. **(B–D)** Variation among individual accessions. All individuals within the clonal variety “BaoxSP07” **(B)** cluster tightly, whereas the seed accession “Baox” **(C)** shows more variation, including one off-type. Importantly, although both **B,C** are supposedly Baox, they do not cluster with each other on the tree. **(D)** The “Wife” accession showed extreme variation, with individual plants scattered across the entire tree. (See Supplementary Figure 2 for more details on individual accessions.)

All 148 remaining female plants were genotyped with genotyping-by-sequencing (GBS), resulting in ∼48,000 markers after filtering (see Methods). Genetic clustering showed some expected patterns, such as the fiber accessions clustering together and clones clustering tightly to each other (Figure 1A). Minor differences among clones are presumably due to a low level of sequencing errors that made it through our filters.

Most of the seed-grown CBD accessions showed little consistency. For example, two accessions named “Baox” (“Baox” and “BaoxSP07,” from 2 different suppliers) showed no real relationship to each other. Most accessions are split across the tree and involve at least two separate clusters. Some of these seem to be the result of a single outlier (e.g., Baox and Kau’XXX), but others include multiple individuals in each cluster (Abacus Early Bird; Abacus Early Bird 2; KauXX, Otto II), and still others are scattered across the entire tree (AbacusxBB1, Berry Blossom, Wife) (Figures 1B–D; Supplementary Figures 2, 3).

To compare these data to prior results, we downloaded public whole-genome sequencing data from 55 *Cannabis sativa* accessions (Lynch et al., 2016) and called SNPs using our same pipeline. This resulted in 8867 quality-filtered SNPs shared between the datasets. Based on these SNPs, we find that CBD-, fiber-, and seed-type accessions cluster with their respective types, regardless of which dataset they originate from. Meanwhile, all the published marijuana-type plants cluster separately (Supplementary Figure 4). These results match prior published data, where CBD-type hemp plants are usually genetically distinct from marijuana (Sawler et al., 2015; Lynch et al., 2016; Schwabe et al., 2019; Grassa et al., 2021).

**FIGURE 2:**
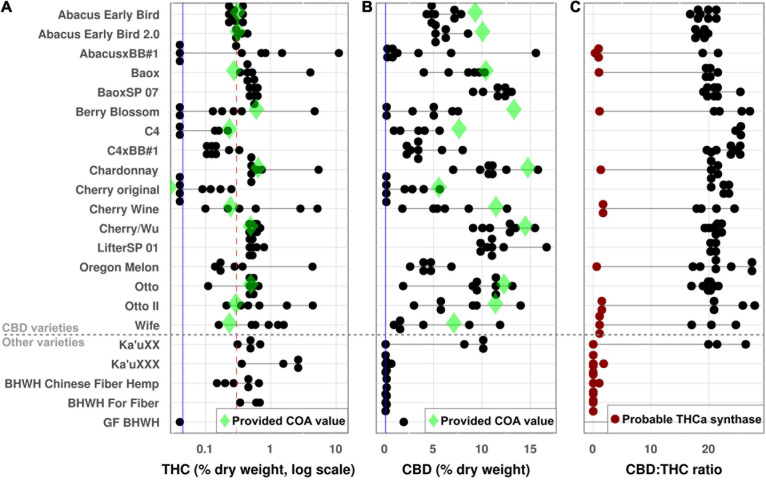
Cannabinoid analysis. THC and CBD were determined for all plants as a percent dry weight of harvested material. Each dot represents a single plant, and horizontal black lines show the range of values for a given accession. Green diamonds show the value provided on the Certificate of Analysis (COA) for each variety, and vertical blue lines show the limit of detection. **(A)** THC values. The red dotted line is maximum allowable value in the United States (0.3%); plants above this line would be considered marijuana. **(B)** CBD. **(C)** CBD:THC ratio. Red dots indicate plants whose measured CBD:THC ratio indicates the presence of at least one active THCa synthase gene.

Of the 148 female plants, eleven had flowers that did not properly develop, leaving 137 flower samples to test for cannabinoid content. THC levels ranged from undetectable up to 11.08% THC by dry weight. Eighty-nine plants produced flowers with more than the legal limit of 0.3% THC, including 15 plants with >1% THC and one with >10% THC (Figure 2A). CBD levels ranged from undetectable (mostly fiber varieties) up to 16.7% dry weight (Figure 2B). Twelve plants produced THC without any CBD, including three of them with >1% THC by dry weight, though all of these were fiber varieties.

Similar to the genetic relationships, most accessions showed little consistency for cannabinoid production. The clones (“BaoxSP 07” and “LifterSP 01”) were generally tightly clustered for both THC and CBD (Figure 2), but seed-grown accessions showed significant variability for both phenotypes. The most concerning ones were several accessions (AbacusxBB1, Berry Blossom, and Cherry original) which were sold as high-CBD lines but had multiple plants with no detectable CBD production, representing a wasted investment for growers. Meanwhile, one plant of “AbacusxBB1” contained >10% THC by dry weight, meaning it is not just legally but functionally a drug-type marijuana plant. Although this chemical variability reflects the genetic variability in accessions, there was not a clear relationship between the two, meaning that the more genetically variable accessions were not also more chemically variable (Supplementary Figure 5).

The ratio of CBD to THC varied from ∼0 (for the plants that produced no CBD) up to ∼28:1 (Figure 2C). Some plants produced no detectable THC (and thus have no ratio), but these plants also had very low levels of CBD (Supplementary Table 2). Independent of genetic data, the CBD:THC ratio can indicate which genes are present in the plant, with ratios of ∼20:1 indicating no active THCa synthase and ratios of ∼2:1 indicating at least one active THCa synthase gene (Toth et al., 2020). Based on their chemical profiles, twenty-eight plants had ratios that indicate the potential presence of a copy of the THCa synthase gene (Figure 2C).

Twelve accessions came with a certificate of analysis (COA), which suppliers use to show what level of cannabinoid production should be expected from the plant. These certificates are important for growers to know that their crop will remain under the 0.3% legal limit for THC, along with estimating the return on investment for CBD. However, most accessions had less CBD than their COA showed, and almost all of them had more THC (Figure 2), both of which could potentially cause issues for commercial growers.

## Discussion

The current federal regulations have created a fine line between legal hemp and illegal marijuana. Inconsistencies in plant genetics can greatly complicate the already complex process of legally growing high-CBD hemp. Although planting clones ensures the highest consistency, many farmers choose to plant seeds because of their much lower cost. This lower cost comes with risk due to inconsistent plant genetics and seed feminization that can make it difficult to produce hemp profitably (Meier and Mediavilla, 1998).

### Seed Feminization

Many farmers have received seeds that were improperly feminized or not feminized at all, resulting in lost revenue and lawsuits (Associated Press, 2019). All feminized product used in this experiment produced only female flowers, although the numbers were too small to draw definite conclusions from Farmers consider a seed lot to be well feminized if less than 1 in 4,000 plants produce male flowers; (personal observation).

### Genetic Relationships

Most accessions tested showed little genetic consistency (Figure 1 and Supplementary Figures 2, 3), which likely explains their phenotypic inconsistency (Figure 2). As expected, clonal accessions were the major exception, though some seed accessions (like Chardonnay) showed good within-accession consistency. This indicates that at least some accessions from some suppliers are reliable, although without extensive testing it is impossible to say which. Conversely, plant accessions with the same name but from different suppliers did not show any genetic clustering (Figures 1A,B and Supplementary Figure 2), meaning that they are actually no more related than any two random accessions. Growers should be careful when purchasing materials; the best approach is probably to just assume that each supplier is selling completely different seed regardless of what they name it. In this way, the naming issues of high-CBD hemp appear to parallel those of marijuana-type Cannabis (Sawler et al., 2015; Schwabe and McGlaughlin, 2019; Vergara et al., 2019).

### CBD Accessions Without CBD

The fact that many CBD accessions contained plants that produced no detectable CBD is concerning (Figure 2A). The most likely reason for this result is that the seed lots contained mixed varieties of plants, some of which lacked the CBDa synthase gene. Copy-number variation for CBDa synthesis genes is extensive in Cannabis (Vergara et al., 2019), and although this is concerning for growers, it has a simple fix: producers should periodically screen their materials via chemical and/or genetic tests to confirm that all of the plants in seed production contain active CBDa synthase. The results of such screening can then be included on the Certificate of Analysis.

### THC Production

The most concerning results from this experiment were the number of plants that produced excessively high levels of THC. The CBDa synthase gene naturally produces low levels of THC (Zirpel et al., 2018), so any plant producing CBD will have some amount of THC. However, plants with as much or more THC production than CBD almost certainly have an active THCa synthase gene (Toth et al., 2020). Eight CBD accessions had at least one plant that showed chemical evidence of an active THCa synthase gene (Figure 2), even though all were supposed to be low-THC varieties. The apparent presence of an active THCa synthase gene in CBD-production lines is very concerning, and the rate was surprisingly high (28 of 121 seed-grown plants, including one plant with > 10% THC). More extensive testing would be needed to see if any of the other CBD accessions also contain plants with active THCa synthase.

All four fiber accessions also showed evidence of active THCa synthase. Since fiber varieties are not grown in such a way to produce large amounts of cannabinoids, their containing THCa synthase is not a problem for fiber growers *per se*. It is, however, of potential concern insofar as any contamination of fiber varieties into CBD accessions (via seed swaps and pollen contamination, etc.) could potentially introduce an active THCa synthase gene into supposedly THC-free varieties. Producers should invest in regularly screening materials for THCa synthase genes in the same way we recommend they test for active CBDa synthase (above) so as to keep their accessions pure. In the meantime, growers may want to invest some time and resources into testing small batches of seeds from different suppliers to identify which ones are the most stable and trustworthy (not to mention high-performing).

### Limits to CBD Production

As previously mentioned, CBDa synthase naturally produces low levels of THC, which explains why almost all the plants tested showed some level of THC (Supplementary Table 2). Even with THC much below CBD production, the plants which produced the highest levels of CBD all exceeded the federal level of 0.3% THC at full maturity. This implies that, with current varieties, there may be a limit to how much CBD a plant can produce while staying below the legal limit of THC. In the long run this limit might be improved by using natural or induced variation in the CBDa synthase gene to select for more specific enzyme variants. For now, however, frequent testing of plants as the flowers mature can help farmers determine when their plants are getting too close to that limit and adjust their harvest times accordingly.

### Certificates of Authenticity

One concerning pattern we noticed was that several COAs were printed so that they show misleadingly low levels of THC. Specifically, they highlighted the low levels of Δ9-THC (the actual psychoactive form) while de-emphasizing THCa (the acid precursor that is decarboxylated into Δ9-THC by heat). United States federal testing guidelines require including both (Agricultural Marketing Service, 2019, 2021), and using the official formula of [total THC] = [Δ9-THC] + 0.877^∗^ [THCa] (Agricultural Marketing Service, 2021), only 7 of the 14 accessions with COAs actually claimed total THC levels below the 0.3% limit. This is a separate issue from how much CBD/THC the plants actually produce (Figure 2), since the COA functions as a decision-making tool for the grower before planting even begins. Some of these COAs may have been issued before the interim final rule that established these guidelines (October 31, 2019) (Agricultural Marketing Service, 2019); if so, one would hope that the companies have updated them with the new guidelines. Nonetheless, growers should pay close attention when ordering materials and ensure that the product information is reported accurately so that they can make the most informed decisions about their product.

## Conclusion

The high-CBD hemp industry is experiencing many growing pains associated with its rapid development in the last 6 years. There are issues with genetic stability, economic viability, and governmental regulations. Despite these issues, the market continues to grow year after year, and interest in this crop continues to expand. With the support that this crop receives from consumers and the support it is beginning to receive from a wide range of researchers, there is a great opportunity for hemp to play an increasingly important role in a wide range of industries. Given the variability we found both among and within accessions, some sort of standardization is needed so that producers can be confident in the material they receive. A good first step would be for suppliers to start using unique names for each of their accessions. Not only will this help clarify the market, it will also allow each company to capitalize on branding their own unique varieties. A more complex but badly needed step is an industry seed-certification process to allow growers to purchase with confidence. Some states are already moving forward with their own seed certifications (HEMP, 2019; Georgia Crop Improvement Association, 2020), but until rigorous, independent verification is implemented across the industry, growers face the prospect of getting a bad lot any time they purchase from a new supplier. Ultimately, these and other changes will need to occur to make the market for high-CBD hemp robust, reliable, and sustainable over the long-term.

## Data Availability Statement

Raw sequencing data is available through the NCBI Sequence Read Archive (BioProject PRJNA707556). Bioinformatic scripts and key intermediate files are available on Github at https://github.com/wallacelab/paper-johnson-hemp-gs.

## Author Contributions

MJ conceived the experiment, performed the bulk of plant growth and cannabinoid assays, and ran the initial bioinformatics analysis. JW assisted with experimental design, oversaw project execution, and performed the final bioinformatic analyses. Both authors wrote and edited the manuscript for publication.

## Conflict of Interest

MJ and JW were involved in the development of new hemp varieties (licensed through the University of Georgia) and received research funds to this end from GaXtracts, where MJ was subsequently employed.

## References

[B1] 2020 US CBD market forecast announcement (2020). Available online at: https://www.brightfieldgroup.com/press-releases/2020-us-cbd-market-forecast-healthy-consolidation (accessed March 4, 2021).

[B2] Agricultural Marketing Service (2019). Establishment of a domestic hemp production program. *Fed. Regist.* 84 58522–58564.

[B3] Agricultural Marketing Service (2021). Establishment of a domestic hemp production program. *Fed. Regist.* 86 5596–5691.

[B4] Alliance D. P. (2014). *The DEA**: Four Decades of Impeding and Rejecting Science.* Available online at: https://drugpolicy.org/resource/dea-four-decades-impeding-and-rejecting-science (accessed June 23, 2021).

[B5] Associated Press (2019). *Oregon Hemp Seed Company Sued for $44 Million After Crops Allegedly Failed. Statesman Journal.* Available online at: https://www.statesmanjournal.com/story/news/2019/09/28/oregon-company-sued-44-million-allegedly-selling-worthless-hemp-seeds/3805696002/ (accessed March 4, 2021).

[B6] Auguie B. (2017). *gridExtra: Miscellaneous Functions for “Grid” Graphics.* Available online at: https://CRAN.R-project.org/package=gridExtra (accessed June 23, 2021).

[B7] Bickel B. Zakharko T. (2016). *RSplitsTree: SplitsTree File Generation and Invoking from R. Github repository.* Available online at: https://github.com/IVS-UZH/RSplitsTree (accessed June 22, 2021).

[B8] Bradbury P. J. Zhang Z. Kroon D. E. Casstevens T. M. Ramdoss Y. Buckler E. S. (2007). TASSEL: software for association mapping of complex traits in diverse samples. *Bioinformatics* 23 2633–2635. 10.1093/bioinformatics/btm308 17586829

[B9] Braich S. Baillie R. C. Jewell L. S. Spangenberg G. C. Cogan N. O. I. (2019). Generation of a comprehensive transcriptome atlas and transcriptome dynamics in medicinal cannabis. *Sci. Rep.* 9:16583. 31719627 10.1038/s41598-019-53023-6 PMC6851104

[B10] Conneely L. J. Mauleon R. Mieog J. Barkla B. J. Kretzschmar T. (2021). Characterization of the *Cannabis sativa* glandular trichome proteome. *PLoS One* 16:e0242633. 10.1371/journal.pone.0242633 33793557 PMC8016307

[B11] Davis T. L. (2019). *argparse: Command Line Optional and Positional Argument Parser.* Available online at: https://CRAN.R-project.org/package=argparse (accessed June 23, 2021).

[B12] de Meijer E. P. M. Bagatta M. Carboni A. Crucitti P. Moliterni V. M. C. Ranalli P. (2003). The inheritance of chemical phenotype in *Cannabis sativa* L. *Genetics* 163 335–346. 10.1093/genetics/163.1.335 12586720 PMC1462421

[B13] de Meijer E. P. M. Hammond K. M. Sutton A. (2009). The inheritance of chemical phenotype in *Cannabis sativa* L. (IV): cannabinoid-free plants. *Euphytica* 168 95–112. 10.1007/s10681-009-9894-7

[B14] Dufresnes C. Jan C. Bienert F. Goudet J. Fumagalli L. (2017). Broad-Scale genetic diversity of cannabis for forensic applications. *PLoS One* 12:e0170522. 10.1371/journal.pone.0170522 28107530 PMC5249207

[B15] Elshire R. J. Glaubitz J. C. Sun Q. Poland J. A. Kawamoto K. Buckler E. S. (2011). A robust, simple genotyping-by-sequencing (GBS) approach for high diversity species. *PLoS One* 6:e19379. 10.1371/journal.pone.0019379 21573248 PMC3087801

[B16] Elsohly M. A. Slade D. (2005). Chemical constituents of marijuana: the complex mixture of natural cannabinoids. *Life Sci.* 78 539–548. 10.1016/j.lfs.2005.09.011 16199061

[B17] Faux A.-M. Draye X. Flamand M.-C. Occre A. Bertin P. (2016). Identification of QTLs for sex expression in dioecious and monoecious hemp (*Cannabis sativa* L.). *Euphytica* 209 357–376. 10.1007/s10681-016-1641-2

[B18] Fleming M. P. Clarke R. C. (1998). Physical evidence for the antiquity of *Cannabis sativa* L. *J. Int. Hemp Assoc.* 5 80–95.

[B19] Georgia Crop Improvement Association (2020). *CROPS ELIGIBLE FOR CERTIFICATION FOR YEAR 2020.* Available online at: http://www.georgiacrop.com/fullpanel/uploads/files/crops-eligible—pvp-level-legend-2020.pdf (accessed June 23, 2021).

[B20] Glass M. Gilleece T. (2019). *Cannabinoids: A Possibility for Pain Management in the Palliative Cancer Pathway. in Annual Radiotherapy Conference (2019).* Available online at: https://pure.ulster.ac.uk/en/publications/%20cannabinoids-a-possibility-for-pain-management-in-the-palliative- (accessed June 23, 2021).

[B21] Grassa C. J. Weiblen G. D. Wenger J. P. Dabney C. Poplawski S. G. Timothy Motley S. (2021). A new Cannabis genome assembly associates elevated cannabidiol (CBD) with hemp introgressed into marijuana. *New Phytol.* 230 1665–1679. 10.1111/nph.17243 33521943 PMC8248131

[B22] HEMP (2019). Available online at: https://seedcert.oregonstate.edu/crop-information/hemp (accessed March 15, 2021).

[B23] Huang Y. Li D. Zhao L. Chen A. Li J. Tang H. (2019). Comparative transcriptome combined with physiological analyses revealed key factors for differential cadmium tolerance in two contrasting hemp (*Cannabis sativa* L.) cultivars. *Ind. Crops Prod.* 140:111638. 10.1016/j.indcrop.2019.111638

[B24] Hurd Y. L. Spriggs S. Alishayev J. Winkel G. Gurgov K. Kudrich C. (2019). Cannabidiol for the reduction of cue-induced craving and anxiety in drug-abstinent individuals with heroin use disorder: a double-blind randomized placebo-controlled trial. *Am. J. Psychiatry* 176 911–922. 10.1176/appi.ajp.2019.18101191 31109198

[B25] Huson D. H. Bryant D. (2006). Application of phylogenetic networks in evolutionary studies. *Mol. Biol. Evol.* 23 254–267. 10.1093/molbev/msj030 16221896

[B26] Hussain A. Calabria-Holley J. Lawrence M. Ansell M. P. Jiang Y. Schorr D. (2019). Development of novel building composites based on hemp and multi-functional silica matrix. *Compos. Part B* 156 266–273. 10.1016/j.compositesb.2018.08.093

[B27] Jenkins C. Orsburn B. (2020). The Cannabis Proteome Draft Map Project. *Int. J. Mol. Sci.* 21:965. 10.3390/ijms21030965 32024021 PMC7037972

[B28] Kassambara A. (2020). *ggpubr: “ggplot2” Based Publication Ready Plots.* Available online at: https://CRAN.R-project.org/package=ggpubr (accessed June 23, 2021).

[B29] Khattab M. M. Dahman Y. (2019). Production and recovery of poly-3-hydroxybutyrate bioplastics using agro-industrial residues of hemp hurd biomass. *Bioprocess Biosyst. Eng.* 42 1115–1127. 10.1007/s00449-019-02109-6 30993443

[B30] Laverty K. U. Stout J. M. Sullivan M. J. Shah H. Gill N. Holbrook L. (2019). A physical and genetic map of *Cannabis sativa* identifies extensive rearrangements at the THC/CBD acid synthase loci. *Genome Res.* 29 146–156. 10.1101/gr.242594.118 30409771 PMC6314170

[B31] Li H. (2011). A statistical framework for SNP calling, mutation discovery, association mapping and population genetical parameter estimation from sequencing data. *Bioinformatics* 27 2987–2993. 10.1093/bioinformatics/btr509 21903627 PMC3198575

[B32] Li H. (2013). Aligning sequence reads, clone sequences and assembly contigs with BWA-MEM. *arXiv* [Preprint]. Available online at: http://arxiv.org/abs/1303.3997 (accessed June 23, 2021).

[B33] Li H.-L. (1974). An archaeological and historical account of cannabis in China. *Econ. Bot.* 28 437–448. 10.1007/bf02862859

[B34] Li S.-Y. Stuart J. D. Li Y. Parnas R. S. (2010). The feasibility of converting *Cannabis sativa* L. oil into biodiesel. *Bioresour. Technol.* 101 8457–8460. 10.1016/j.biortech.2010.05.064 20624607

[B35] Liu J. Qiao Q. Cheng X. Du G. Deng G. Zhao M. (2016). Transcriptome differences between fiber-type and seed-type *Cannabis sativa* variety exposed to salinity. *Physiol. Mol. Biol. Plants* 22 429–443. 10.1007/s12298-016-0381-z 27924117 PMC5120038

[B36] Lubell J. D. Brand M. H. (2018). Foliar sprays of silver thiosulfate produce male flowers on female hemp plants. *Horttechnology* 28 743–747. 10.21273/horttech04188-18

[B37] Lynch R. C. Vergara D. Tittes S. White K. Schwartz C. J. Gibbs M. J. (2016). Genomic and chemical diversity in Cannabis. *CRC Crit. Rev. Plant Sci.* 35 349–363.

[B38] McGarvey P. Huang J. McCoy M. Orvis J. Katsir Y. Lotringer N. (2020). *De novo* assembly and annotation of transcriptomes from two cultivars of *Cannabis sativa* with different cannabinoid profiles. *Gene* 762:145026. 10.1016/j.gene.2020.145026 32781193

[B39] Meier C. Mediavilla V. (1998). Factors influencing the yield and the quality of hemp essential oil. *J. Int. Hemp Assoc.* 5 16–20.

[B40] Merlin M. D. (2003). COVER ARTICLE: archaeological evidence for the tradition of psychoactive plant use in the old world. *Econ. Bot.* 57 295–323. 10.1663/0013-0001(2003)057[0295:aeftto]2.0.co;2

[B41] Oksanen J. Blanchet F. G. Friendly M. Kindt R. Legendre P. McGlinn D. (2020). *vegan: Community Ecology Package.* Available online at: https://CRAN.R-project.org/package=vegan (accessed June 23, 2021).

[B42] Perucca E. (2017). Cannabinoids in the Treatment of Epilepsy: Hard Evidence at Last? *J. Epilepsy Res.* 7 61–76. 10.14581/jer.17012 29344464 PMC5767492

[B43] Ram H. Y. M. Mohan Ram H. Y. Jaiswal V. S. (1972). Induction of male flowers on female plants of *Cannabis sativa* by gibberellins and its inhibition by abscisic acid. *Planta* 105 263–266. 10.1007/bf00385397 24477812

[B44] Russo E. B. (2007). History of cannabis and its preparations in saga, science, and sobriquet. *ChemInform* 4 1614–1648. 10.1002/chin.200747224 17712811

[B45] S. 2667 (115th): Hemp farming act of 2018 (2018). Available online at: https://www.govtrack.us/congress/bills/115/s2667 (accessed March 4, 2021).

[B46] Sakamoto K. Akiyama Y. Fukui K. Kamada H. Satoh S. (1998). Characterization; Genome Sizes and Morphology of Sex Chromosomes in Hemp (*Cannabis sativa* L.). *CYTOLOGIA* 63 459–464. 10.1508/cytologia.63.459

[B47] Sassoni E. Manzi S. Motori A. Montecchi M. Canti M. (2014). Novel sustainable hemp-based composites for application in the building industry: physical, thermal and mechanical characterization. *Energy Build.* 77 219–226. 10.1016/j.enbuild.2014.03.033

[B48] Sawler J. Stout J. M. Gardner K. M. Hudson D. Vidmar J. Butler L. (2015). The genetic structure of marijuana and hemp. *PLoS One* 10:e0133292. 10.1371/journal.pone.0133292 26308334 PMC4550350

[B49] Schwabe A. L. Hansen C. J. Hyslop R. M. McGlaughlin M. E. (2019). Research grade marijuana supplied by the National Institute on Drug Abuse is genetically divergent from commercially available Cannabis. *bioRxiv* [Preprint]. 10.1101/592725

[B50] Schwabe A. L. McGlaughlin M. E. (2019). Genetic tools weed out misconceptions of strain reliability in *Cannabis sativa*: implications for a budding industry. *J. Cannabis Res.* 1:3. 10.1186/s42238-019-0001-1 33526091 PMC7815053

[B51] Sirikantaramas S. Morimoto S. Shoyama Y. Ishikawa Y. Wada Y. Shoyama Y. (2004). The gene controlling marijuana psychoactivity: molecular cloning and heterologous expression of Delta1-tetrahydrocannabinolic acid synthase from *Cannabis sativa* L. *J. Biol. Chem.* 279 39767–39774. 15190053 10.1074/jbc.M403693200

[B52] Taura F. Morimoto S. Shoyama Y. Mechoulam R. (1995). First direct evidence for the mechanism of.D*ELTA.*1-tetrahydrocannabinolic acid biosynthesis. *J. Am. Chem. Soc.* 117 9766–9767. 10.1021/ja00143a024

[B53] Taura F. Sirikantaramas S. Shoyama Y. Yoshikai K. Shoyama Y. Morimoto S. (2007). Cannabidiolic-acid synthase, the chemotype-determining enzyme in the fiber-type *Cannabis sativa*. *FEBS Lett.* 581 2929–2934. 10.1016/j.febslet.2007.05.043 17544411

[B54] Toth J. A. Stack G. M. Cala A. R. Carlson C. H. Wilk R. L. Crawford J. L. (2020). Development and validation of genetic markers for sex and cannabinoid chemotype in *Cannabis sativa* L. *GCB Bioenergy* 12 213–222. 10.1111/gcbb.12667

[B55] Turner J. C. Hemphill J. K. Mahlberg P. G. (1981). Interrelationships of glandular trichomes and cannabinoid content. II. Developing vegetative leaves of *Cannabis sativa* L. (Cannabaceae). *Bull. Narc.* 33 63–71. 6279216

[B56] van Bakel H. Stout J. M. Cote A. G. Tallon C. M. Sharpe A. G. Hughes T. R. (2011). The draft genome and transcriptome of *Cannabis sativa*. *Genome Biol.* 12:R102. 22014239 10.1186/gb-2011-12-10-r102 PMC3359589

[B57] van den Broeck H. C. Maliepaard C. Ebskamp M. J. M. Toonen M. A. J. Koops A. J. (2008). Differential expression of genes involved in C1 metabolism and lignin biosynthesis in wooden core and bast tissues of fibre hemp (*Cannabis sativa* L.). *Plant Sci.* 174 205–220. 10.1016/j.plantsci.2007.11.008

[B58] Vergara D. Huscher E. L. Keepers K. G. Givens R. M. Cizek C. G. Torres A. (2019). Gene copy number is associated with phytochemistry in *Cannabis sativa*. *AoB Plants* 11:lz074. 10.1093/aobpla/plz074 PMC6986684 32010439

[B59] Vergara D. Huscher E. L. Keepers K. G. Pisupati R. Schwabe A. L. McGlaughlin M. E. (2021). Genomic evidence that governmentally produced *Cannabis sativa* poorly represents genetic variation available in state markets. *bioRxiv* [Preprint]. 10.1101/2021.02.13.431041 PMC8476804 34594346

[B60] Wang L.-G. Lam T. T.-Y. Xu S. Dai Z. Zhou L. Feng T. (2020). Treeio: an R Package for phylogenetic tree input and output with richly annotated and associated data. *Mol. Biol. Evol.* 37 599–603. 10.1093/molbev/msz240 31633786 PMC6993851

[B61] Weiblen G. D. Wenger J. P. Craft K. J. ElSohly M. A. Mehmedic Z. Treiber E. L. (2015). Gene duplication and divergence affecting drug content in *Cannabis sativa*. *New Phytol.* 208 1241–1250. 10.1111/nph.13562 26189495

[B62] Wickham H. (2016). *ggplot2: Elegant Graphics for Data Analysis.* Available online at: https://ggplot2.tidyverse.org (accessed June 23, 2021).

[B63] Wickham H. Averick M. Bryan J. Chang W. McGowan L. D. François R. (2019). Welcome to the tidyverse. *J. Open Source Softw.* 4:1686. 10.21105/joss.01686

[B64] Wretfors C. Cho S.-W. Hedenqvist M. S. Marttila S. Nimmermark S. Johansson E. (2009). Use of industrial hemp fibers to reinforce wheat gluten plastics. *J. Polym. Environ.* 17:259. 10.1007/s10924-009-0147-6

[B65] Yu G. (2020). Using ggtree to visualize data on tree-like structures. *Curr. Protoc. Bioinformatics* 69:e96. 32162851 10.1002/cpbi.96

[B66] Zirpel B. Kayser O. Stehle F. (2018). Elucidation of structure-function relationship of THCA and CBDA synthase from *Cannabis sativa* L. *J. Biotechnol.* 284 17–26. 10.1016/j.jbiotec.2018.07.031 30053500

