# Supplementary material for: Genomic and Chemical Diversity of Commercially Available High-CBD Industrial Hemp Accessions
Source: Front Genet. 2021 Jul 7;12:682475. doi: 10.3389/fgene.2021.682475 (PMC8293613; doi:10.3389/fgene.2021.682475)
Supplement: Supplementary file 1 [file Data_Sheet_1.PDF]

Supplemental Data

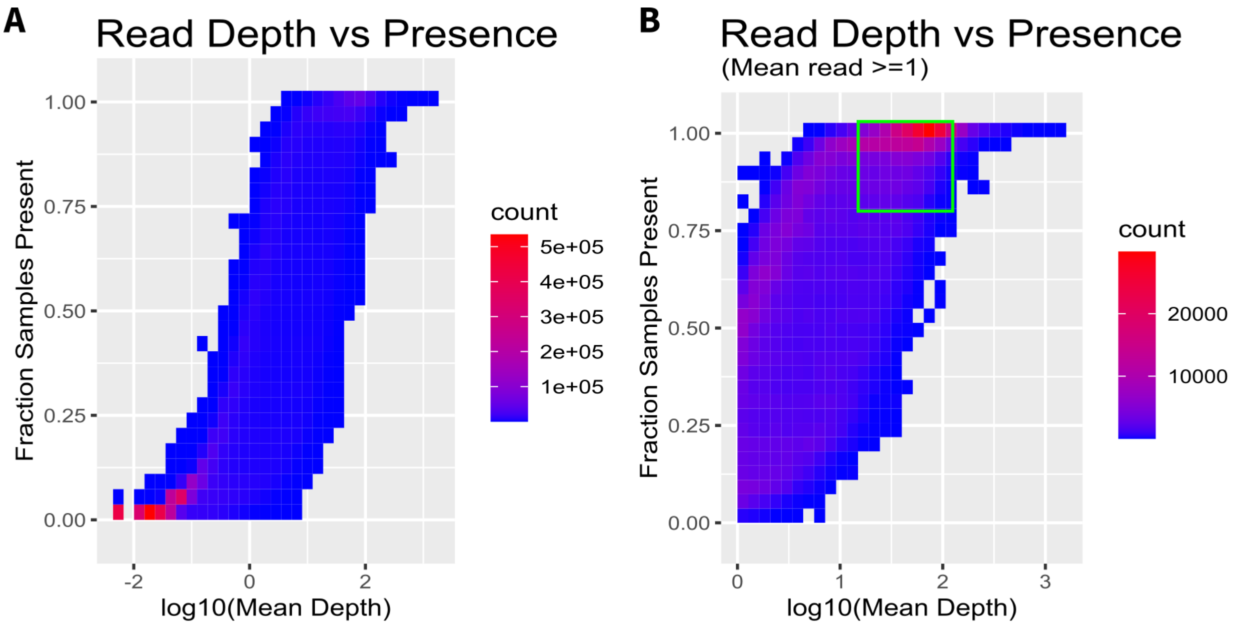

Supplemental Figure S1 - Read Depth Cutoff. All sites in the raw, aligned genotyping data were graphed based on their mean depth per sample (x axis) versus the number of samples with a genotype call (y axis). (A) Raw genotyping data contains a large number of sites with low depth that are only present in a small number of samples (red patch in the lower-left); these are likely misalignments. (B) After removing sites with mean depth <=1 (0 on the log scale), a secondary cluster of true sites with high depth and high coverage is visible. The green box indicates which sites were chosen for downstream analysis.

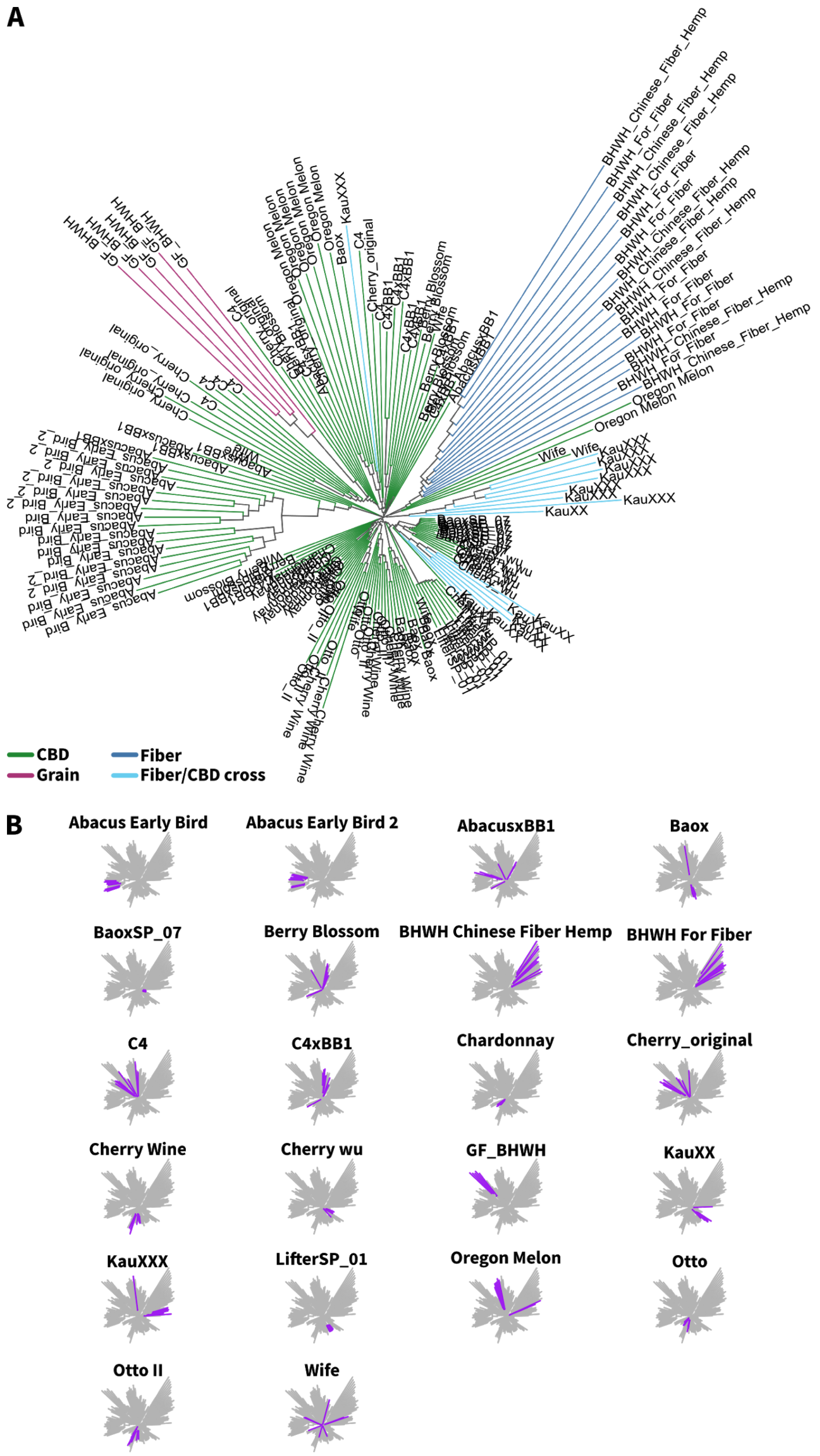

Supplemental Figure S2 - Cladograms. (A) Neighbor-joining tree of all genotyped plants with labeled tips. (B) The same tree as in (A), but with each accession highlighted. Some accessions are tightly clustered, while others are scattered across the tree. Compare Figure 1 in the main text.

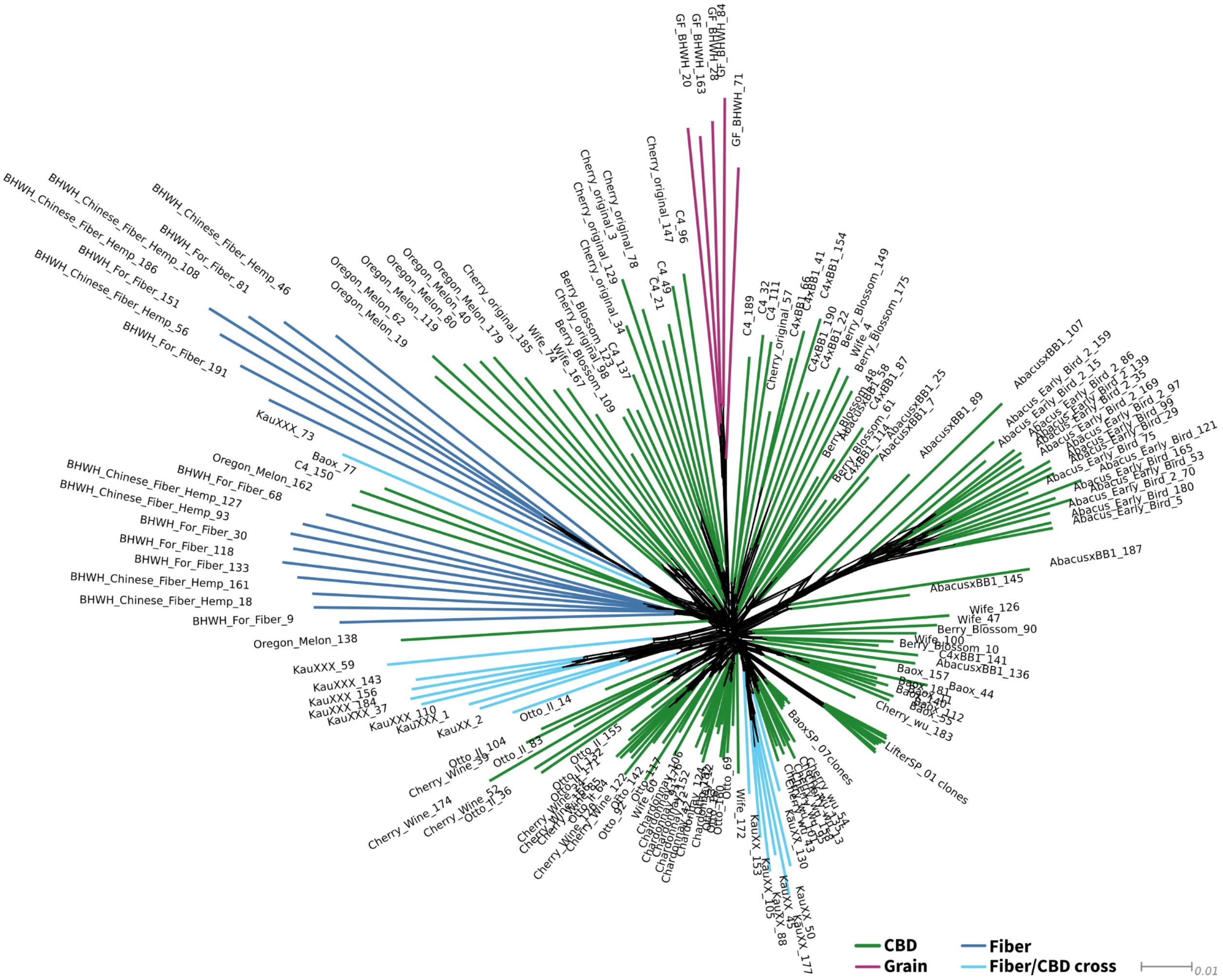

Supplemental Figure S3 - Neighbor-Net of Plant Relationships. A Neighbor-Net of genetic relationships among the samples was created using SpitsTree 4 (Huson and Bryant 2006). Neighbor-nets allow for reticulation, meaning the “webbing” in the cladogram represents ambiguity and can result in a different ordering. Comparing to Supplemental Figure S2 (neighbor-joining tree) indicates that the majority of the clusters are consistent between the two methods, with some minor changes (e.g., a small number of CBD varieties splitting the fiber varieties into two groups.)

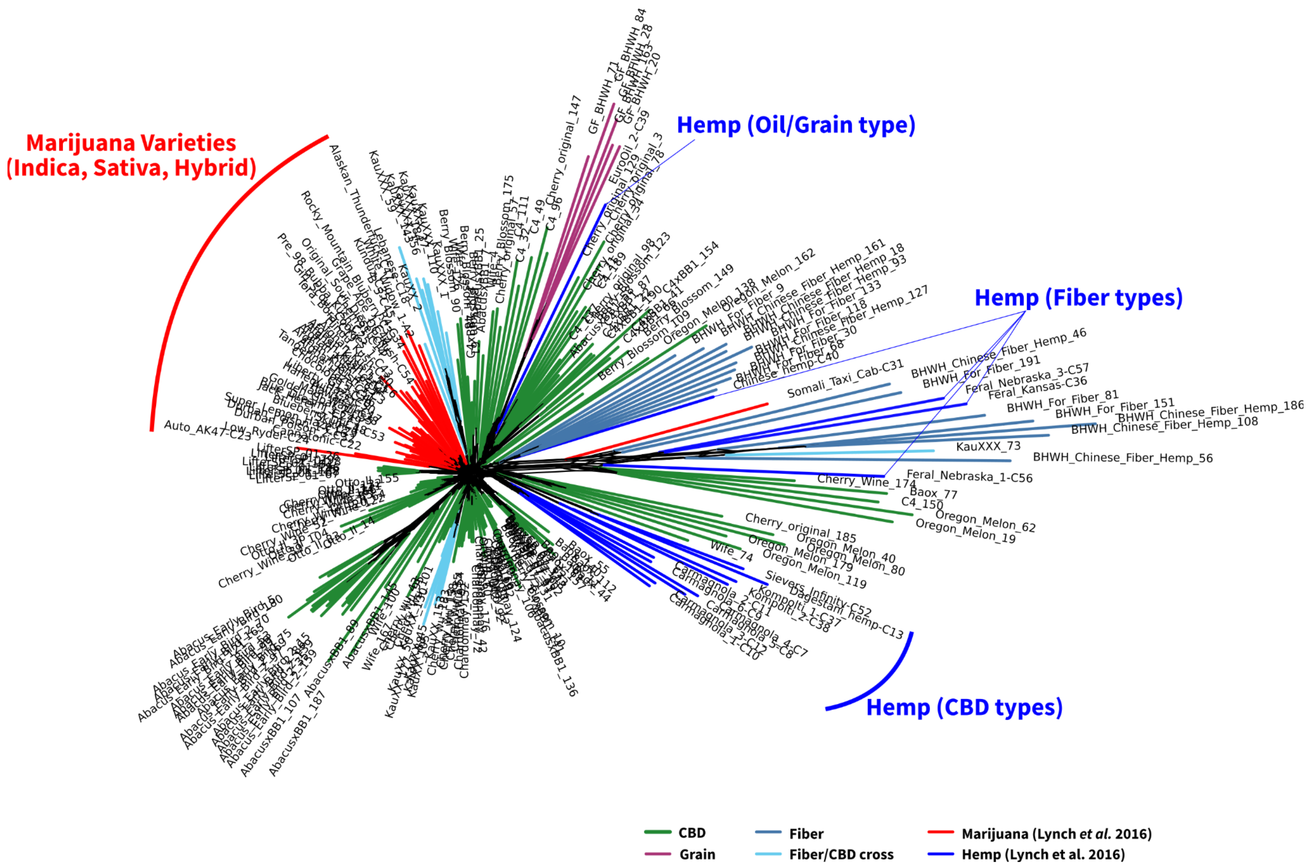

Supplemental Figure S4 - Relationship to Prior Data. Whole-genome sequencing data of 55 previously published *C. sativa* accessions (Lynch et al. 2016) was run through the same SNP-calling pipeline as the current study and merged with the current data, then a Neighbor-Net of genetic relationships among the samples was created using SpitsTree 4 (Huson and Bryant 2006). (Compare Figure S3.) Prior fiber, grain/oil, and CBD-type hemp accessions cluster with the same type of accessions from the current study, while all but one of the marijuana-type varieties cluster separately. (The one exception is likely a mislabel or technical error.)

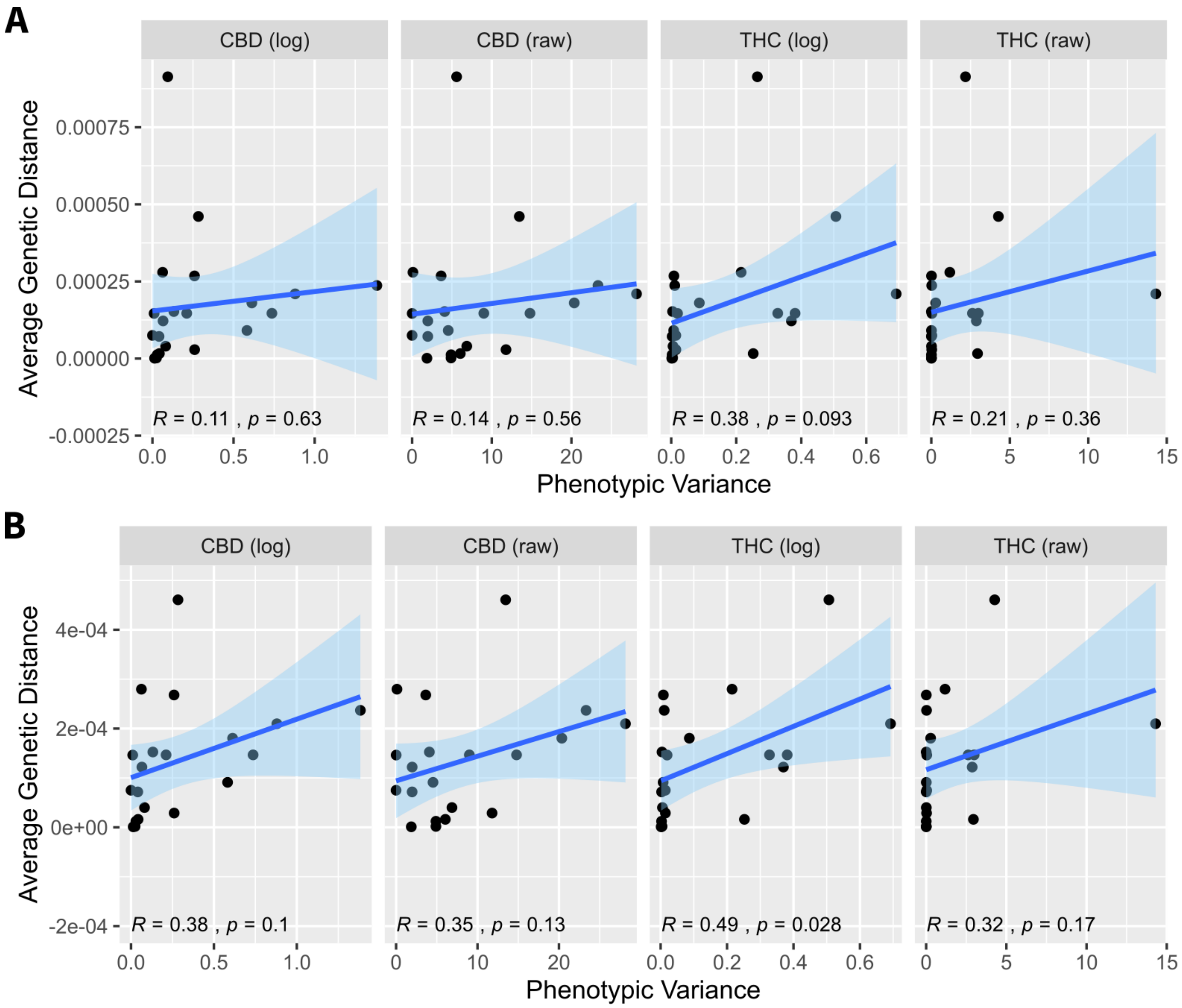

Supplemental Figure S5 - Genetic Versus Chemical Diversity. The genetic variability of each accession was determined by calculating the average genetic distance among all accessions and comparing that to the variance of both raw and log-transformed CBD and THC measurements. Only individuals with both genetic and chemical data were used in these analyses, and log+1 transformation was applied to retain individuals with no detectable CBD or THC. No chemical variability is significantly correlated with genetic diversity when all accessions are included (A), though the log-transformed THC levels become significant when one outlier (Baox) is excluded (B; p=0.028).

Supplemental Table S1: Number of male plants per accession

Line	Type	# Males (of 8)
Abacus_Early_Bird	Feminized	0
Abacus_Early_Bird_2.0	Feminized	0
AbacusxBB#1	Feminized	0
Baox	Non-Feminized	2
BaoxSP_07	Clone	0
Berry Blossom	Feminized	0
BHWH_Chinese_Fiber_Hemp	Non-Feminized	2
BHWH_For_Fiber	Non-Feminized	4
C4	Non-Feminized	1
C4xBB#1	Feminized	0
Chardonnay	Feminized	0
Cherry (original)	Feminized	0
Cherry Wine	Non-Feminized	2
Cherry/Wu	Non-Feminized	1
GF_BHWH	Non-Feminized	3
Ka'uXX	Non-Feminized	4
Ka'uXXX	Non-Feminized	4
LifterSP_01	Clone	0
Oregon Melon	Non-Feminized	2
Otto	Feminized	0
Otto II	Non-Feminized	1
Wife	Feminized	0
